# Neo-BCV: A Novel Bacterial Liquid Complex Vaccine for Enhancing Dendritic Cell-Mediated Immune Responses Against Lung Cancer

**DOI:** 10.3390/vaccines13010064

**Published:** 2025-01-13

**Authors:** Zilong Zhu, Zhuze Chu, Fei Fei, Chenxi Wu, Zhengyue Fei, Yuxia Sun, Yun Chen, Peihua Lu

**Affiliations:** The Affiliated Wuxi People’s Hospital of Nanjing Medical University, Wuxi People’s Hospital, Wuxi Medical Center, Nanjing Medical University, Wuxi 214023, China; zhuzilong2022@126.com (Z.Z.); chuzhuze2022@126.com (Z.C.); ffyuhh1998@126.com (F.F.); wcxsgcjdqg@126.com (C.W.); sugaaaa930309@126.com (Z.F.); ahsunyuxia@126.com (Y.S.)

**Keywords:** tumor vaccines, dendritic cells, JAK2-STAT3 signaling, non-small cell lung cancer

## Abstract

Background: In the past decade, immunotherapy has become a major choice for the treatment of lung cancer, yet its therapeutic efficacy is still relatively limited due to the various immune escape mechanisms of tumors. Based on this, we introduce Neo-BCV, a novel bacterial composite vaccine designed to enhance immune responses against lung cancer. Methods: We investigated the immune enhancing effect of Neo-BCV through in vivo and in vitro experiments, including flow cytometry, RNA-seq, and Western blot. Results: We have demonstrated that Neo-BCV can promote Dendritic cells (DCs) maturation and induce DCs differentiation into pro-inflammatory subgroups, significantly enhancing cytotoxic T lymphocyte (CTL)-mediated anti-tumor responses. Transcriptome sequencing revealed that Neo-BCV exerts its effects by specifically inhibiting the JAK2-STAT3 signaling pathway, a crucial regulator of cancer progression, metabolism, and inflammation. Moreover, Neo-BCV significantly improved the immune microenvironment in both tumor and spleen tissues without inducing notable toxic effects in major organs. Conclusions: These findings highlight Neo-BCV’s potential as a safe and effective therapeutic strategy, offering a novel avenue for clinical translation in lung cancer immunotherapy.

## 1. Introduction

Lung cancer is one of the cancers with the highest morbidity and mortality in the world. The traditional clinical treatment of cancer, including surgical resection and chemoradiotherapy, often has a poor prognosis. In the past few years, immunotherapy has made great progress in treating cancer [1,2]. Especially immune checkpoint inhibitors (ICIs) have achieved great success and widespread recognition [2,3]. However, the effectiveness of immunotherapy for advanced lung cancer remains relatively limited [4]. In the search for new and more effective treatments, we turned our attention to tumor vaccines.

Bacteria have a wide range of applications in cancer immunotherapy. The unique characteristics of bacteria, such as targeting, immunogenicity, and natural toxicity, make them excellent therapeutic drugs or carriers [5,6,7]. More than a hundred years ago, Coley’s Toxin developed by William B. Coley successfully cured multiple patients with different types of advanced cancer, achieving impressive therapeutic effects. Multiple bacteria have been shown to activate innate and adaptive immune responses, thereby recognizing and attacking cancer cells. For instance, *Clostridium* can target hypoxic tumor environments and secrete enzymes and toxins, then activating the immune system and inducing tumor cell apoptosis [8,9]; *Corynebacterium diphtheriae* produces a potent diphtheria toxin (DT), a single molecule of which can cause toxicity as soon as it enters a tumor cell [10,11]; *Salmonella* can accumulate in tumor sites, proliferate, and activate immunogenic responses, thereby inducing neutrophil and dendritic cell (DC) infiltration to attack tumors [12,13]. Compared to using a single bacterium, the combination of multiple bacterial strains can enhance efficacy through different mechanisms. In addition, the use of immune adjuvants and nanocarriers enables bacterial therapy to exert better anti-cancer effects [14,15]. β-glucan can bind to β-glucan receptors on the surface of immune cells and activate an anti-tumor immune response [16]. Meanwhile, β-glucan-coated liposomes can further enhance targeted delivery and improve liposome stability [17,18,19].

DCs play a crucial role in initiating T cell-mediated anti-tumor immunity. In solid tumors, DCs are responsible for capturing and presenting antigens, transporting tumor antigens to draining lymph nodes to activate T cells [20]. Additionally, mature DCs can further promote T cell activation through the production of co-stimulatory molecules and immunogenic cytokines, such as CD80/CD86, TNF-α, and IFN-γ [21]. This antigen cross-presentation enhances the efficacy of cancer vaccines. DCs can present intracellular antigens via MHC I molecules to activate CD8^+^ cytotoxic T lymphocytes (CTLs), while MHC II molecules present extracellular antigens to preferentially activate CD4^+^ T cells [22,23]. Overall, DCs are crucial target cells in vaccine-based therapies.

Based on the previous bacterial liquid vaccine of Academician Kecheng Xu, this study focuses on the development of a novel bacterial liquid composite vaccine (Neo-BCV), consisting of heat-inactivated bacterial strains and adjuvants, including *diphtheria*, *pertussis*, *tetanus*, *typhoid-paratyphoid*, and *Staphylococcus aureus* [24]. Our research has shown that Neo-BCV can exert anti-tumor immune effects by activating and inducing DCs to differentiate into pro-inflammatory phenotypes. Therefore, this study aims to explore how Neo-BCV promotes the activation of dendritic cells, thereby inducing anti-tumor immune responses, and provide insights into the potential application of bacterial composite vaccines in lung cancer immunotherapy.

## 2. Materials and Methods

### 2.1. Materials

GM-CSF and IL-4 were purchased from SinoBiological; LPS was from MedChemExpress (Monmouth Junction, NJ, USA). Mouse lung adenocarcinoma Lewis cells were obtained from Wuhan Pricella Biotechnology (Wuhan, China). Cell culture materials were from Gibco (Grand Island, NY, USA), such as DMEM medium, fetal bovine serum (FBS), and penicillin-streptomycin double antibody. The digestion kit was purchased from Miltenyi Biotec (Bergisch Gladbach, Germany). TRIzol, fixation, and permeabilization kits were obtained from Invitrogen (Carlsbad, CA, USA). Tribromoethanol anesthetic was from Jitian Biotechnology (Jilin, China). Immunohistochemical materials were purchased from Servicebio (Wuhan, China), such as histochemical pen, tissue fixative, and DBA colorimetric solution. All immunohistochemical antibodies were purchased from Proscientia (San Diego, CA, USA). All flow cytometry antibodies used were purchased from Biolegend (San Diego, CA, USA). DNase I was from TaKaRa (Shiga, Japan). The primary antibody for Western Blot was purchased from Chengdu Zhengneng Company (Chengdu, China); the secondary antibody for Western Blot was purchased from Santa Cruz Biotechnology (Dallas, TX, USA). The ECL color development kit was purchased from Thermo Scientific (Waltham, MA, USA). SDS-PAGE electrophoresis gel preparation kit A, B, BCA protein kit, and protein loading buffer were purchased from Vazyme Biotechnology (Nanjing, China). PVDF membrane was purchased from Keygen Biotechnology (Nanjing, China).

### 2.2. Composition and Preparation of Neo-BCV

The BCV included 6 kinds of heat-inactivated bacteria, namely (1) Pertussis bacillus (concentration 9 billion/mL), (2) Diphtheria bacillus endotoxin (concentration 20 Lf/mL), (3) Tetanus bacillus endotoxin (concentration 5 Lf/mL), (4) Typhoid bacillus (concentration of 300 million/mL), (5) Bacillus paratyphoid A/B (concentration 150 million/mL each), and (6) 10% *Staphylococcus aureus* solution (concentration 1 billion/mL). Strains were purchased from the National Institutes for Food and Drug Control (Silver Spring, MD, USA). Zhejiang Weixin Biological Pharmaceutical Co., Ltd. (Dongyang, China), was entrusted with technical service and culture of bacterial media. In addition, 0.2 g/mL glucan was added as an adjuvant. BCV had been proven to have no risk of clinical infection.

### 2.3. BMDC Culture

Harvest the femurs and tibias of the mice, flush, and filter to collect the bone marrow suspension. Add ammonium chloride red blood cell lysis buffer to remove red blood cells, then resuspend the cells in RPMI 1640 medium (with 10% FBS). After centrifugation, resuspend the cells in complete RPMI 1640 medium (with 20 ng/mL GM-CSF, 10 ng/mL IL-4, and 10% FBS), and adjust the cell concentration to 0.5–1 × 10^6^/mL. Then, plate the cells in a 24-well plate and culture for 2 days. Replace 3/4 of the medium with fresh RPMI 1640 complete medium, removing the non-adherent cells. After 2 more days, replace half of the medium. On day 7, collect the floating and loosely adherent BMDCs.

### 2.4. Detection of BMDC Maturity Level by Flow Cytometry

Collect BMDCs induced for 7 days and divide them into three groups: a negative control group, an LPS-positive control group, and the Neo-BCV experimental group. The control group remained untreated, while the LPS group received 1 μg/mL LPS, and the Neo-BCV group received 1 μL/mL Neo-BCV. After 48 h of incubation, collect the cells and wash them twice with PBS. Resuspend the cells in PBS containing antibodies (PE-Cyanine7 anti-CD86, FITC anti-CD11c, PE anti-MHC-II), incubate on ice in the dark for 30 min, wash twice, then resuspend in 500 μL PBS for flow cytometry analysis.

### 2.5. Animal Models and Anti-Tumor Responses

All animal studies were conducted at the Wuxi People’s Hospital Lung Transplantation Animal Laboratory, and the experimental protocol was approved by the Laboratory Animal Management Committee of Nanjing Medical University. Lewis lung cancer (LLC) from iCell ((iCell-0078a) is a cell line established from the lungs of C57BL mice, which is widely used to construct tumor transplantation models. Prepare a single-cell suspension of LLC (1 × 10^7^ cells/mL) and inject 0.1 mL subcutaneously into mice, 1 cm away from the right axilla. Once tumors reached 60–70 mm^3^, randomly divide the mice into two groups: a PBS control group and a Neo-BCV group, with six mice per group. The drug was administered starting on the second day after grouping, and mice were subcutaneously injected with a dose of 0.4 mL every 3 days for a total of 4 injections. Before each treatment, measure the body weight and tumor volume (V) of the mice, with the formula for V being (length × width^2^)/2. For ethical considerations, euthanasia was administered when tumor volume reached 1000 mm^3^ or if mice exhibited signs such as lateral recumbency, cachexia, lack of response to stimuli, or significant weight loss. After the final treatment, mice were anesthetized with tribromoethanol (0.2 mL/20 g) and euthanized by cervical dislocation. Tumor, heart, liver, spleen, lung, and kidney tissues were quickly collected, photographed, and tumor weight measured.

### 2.6. Tumor and Spleen Tissue Flow Cytometry

Cut the tumor into small pieces, digest it with digestive solution, and filter it through a 200-mesh screen to obtain a single-cell suspension of tumor cells. Grind the spleen on a 200-mesh nylon mesh and use red blood cell lysis buffer to remove red blood cells, resulting in a spleen single-cell suspension. Count the cells and adjust the concentration to 1 × 10^7^ cells/mL. Then prepare two antibody mixes: mix-1 (CD45-Percp, CD3-Apc-fire750, CD4-FITC, CD8-BV605) and mix-2 (CD45-Percp, CD3-Apc-fire750, CD11b-Pe-Cy7, CD103-BV786, CD11c-BV650, IA-IE-PE, CD83-APC). Respectively, pipette 100 μL of sample into each of the two flow tubes. Add mix-1 to one tube and mix-2 to the other, then mix thoroughly and incubate at 4 °C in the dark for 30 min. Wash twice with PBS and resuspend in 300 μL PBS. For intracellular staining, follow the instructions of the fixation and permeabilization kits (Invitrogen). Use the flow cytometry tube containing mix-1, then add Granzyme B and Perforin antibodies, and incubate at 4 °C in the dark for 40 min. Wash twice with PBS, then resuspend in 300 μL PBS. All samples are analyzed using a flow cytometer (BD LSRFortessa, BD, Franklin Lakes, NJ, USA), and data are processed with FlowJo Version 10.5.0 software.

### 2.7. Immunohistochemical Staining of Spleen

The spleen was fixed with 4% paraformaldehyde for 24 h, embedded in paraffin, and sectioned. The sections were deparaffinized and rehydrated, followed by heat-induced antigen retrieval to expose the target protein. Endogenous peroxidase activity was quenched using a peroxidase inhibitor. After blocking with blocking solution, the tissue was incubated with primary and secondary antibodies, and DAB was used for colorimetric detection. The nuclei were counterstained with hematoxylin. Finally, the sections were observed and imaged under a microscope, and image analysis was performed using image J (Version 2.5).

### 2.8. Tumor Tissue RNA Extraction and Sequencing

Total RNA was extracted from the tumor tissues using TRIzol^®^ Reagent according to the manufacturer’s instructions (Invitrogen), and genomic DNA was removed using DNase I (TaKara). Then RNA quality was determined using the 2100 Bioanalyser (Agilent, Santa Clara, CA, USA) and quantified using the ND-2000 (NanoDrop Technologies, Wilmington, NC, USA). A high-quality RNA sample (OD260/280 = 1.8–2.2, OD260/230 ≥ 2.0, RIN ≥ 6.5, 28S:18S ≥ 1.0, >10 μg) was used to construct a sequencing library. RNA-seq transcriptome libraries were prepared following TruSeqTM RNA sample preparation Kit from Illumina (San Diego, CA, USA), using 1 μg of total RNA. Shortly, messenger RNA was isolated with polyA selection by oligo (dT) beads and fragmented using fragmentation buffer. cDNA synthesis, end repair, A-base addition, and ligation of the NGS-indexed adaptors were performed. Libraries were then size selected for cDNA target fragments of 200–300 bp on 2% Low Range Ultra Agarose followed by PCR amplification using Phusion DNA polymerase (NEB) for 15 PCR cycles. After quantified by TBS380, Paired-end libraries were sequenced (150 bp × 2, Shanghai BIOZERON Co., Ltd., Shanghai, China).

### 2.9. Western Blot

Take about 0.1 g of the tissue sample and add 1 mL of RIPA lysis buffer (adding 10 μL of PMSF to every 1 mL of lysis buffer). Use a tissue homogenizer to homogenize the sample. Incubate the homogenized tissue at 4 °C for 30 min, then centrifuge at 12,000 rpm for 10 min at 4 °C to collect the supernatant. After quantifying the protein concentration of the supernatant using the BCA method, store it at −80 °C for long-term preservation. Based on the BCA protein quantification results, take approximately 80 μg of total protein and add an appropriate amount of 5× protein loading buffer. Mix well and denature the sample in boiling water for 10 min. Briefly centrifuge, then load the sample into the gel wells. Use 140 V electrophoresis until it just runs out of the separation gel and stop the electrophoresis. After electrophoresis, remove the gel and place it in the prepared 1× transfer buffer to equilibrate for 20 min. Cut the PVDF membrane and filter paper to appropriate sizes, activate the PVDF membrane with methanol, and then place the PVDF membrane and filter paper into the transfer buffer to equilibrate for 20 min. Arrange them in the order of negative electrode-filter paper-gel-PVDF membrane-filter paper-positive electrode. Perform the transfer at a constant current of 200 mA for 1 h. Block the membrane using a 5% non-fat milk solution prepared with 1× TBST. According to the antibody instructions (1:1000), add 6 μL of the primary antibody and incubate overnight at 4 °C on a shaker. Then wash the membrane three times with 1× TBST, 10 min each time. After washing, place the membrane into the diluted secondary antibody solution (1:3000) and incubate at room temperature on a shaker for 1 h, followed by three washes with 1× TBST, 10 min each time. Finally, prepare an appropriate amount of ECL working solution according to the instructions of the ECL detection kit. Drain the liquid from the PVDF membrane, evenly apply the prepared ECL working solution to cover the entire membrane, and incubate at room temperature for 2 min. Then remove the excess solution and place the membrane into the automatic chemiluminescence imaging system for imaging and photographing. Use the Gelpro32 software (Version 4.0.00.001) to perform grayscale analysis on the bands.

### 2.10. H&E Staining

Deparaffinize the tissue sections in water, then immerse them in hematoxylin solution to stain the nuclei. After washing off the excess stain, use eosin solution to stain the cytoplasm. Dehydrate and clear the sections with graded alcohol and xylene, then mount the slides. Finally, observe the sections under a microscope and capture images for further analysis.

### 2.11. Statistical Analysis

Data were analyzed in GraphPad Prism version 8.0 (GraphPad Software). One-way ANOVA, paired and unpaired t test were applied to determine statistical significance between groups. *p <* 0.05 was considered statistically significant.

## 3. Results

### 3.1. Neo-BCV Promotes BMDCs Maturation In Vitro

**Figure 1:**
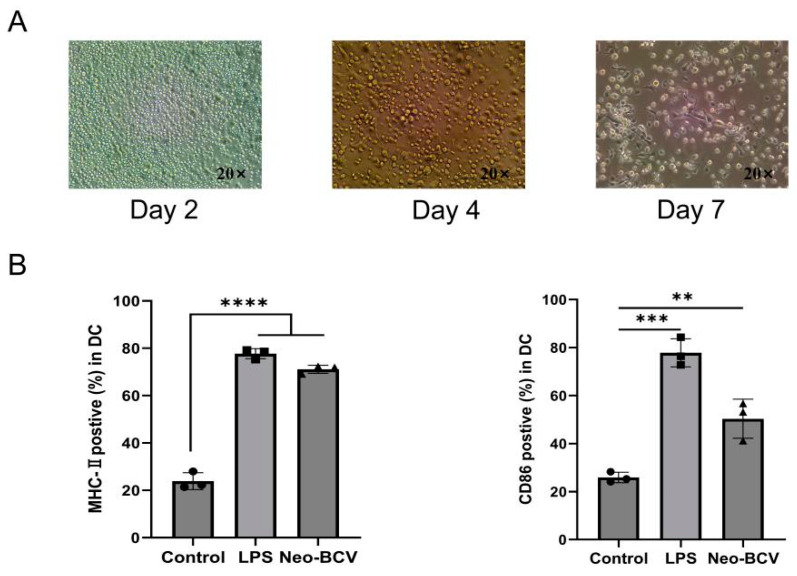
The effect of Neo-BCV on BMDC maturation in vitro. (**A**) Morphology of BMDCs under light microscope. (**B**) Flow cytometry was used to detect the expression levels of CD86 and MHC-II on the surface of DCs, along with a semi-quantitative statistical chart. ** *p* < 0.01, *** *p* < 0.001, **** *p* < 0.0001.

We first extracted DCs from the bone marrow of mice (BMDCs, Bone Marrow-Derived Dendritic Cells) and cultured them for 7 days as shown in Figure 1A. Then collect DCs and divide them into three groups, each treated with different drugs (Figure 1B). Compared with the control group, the expression levels of CD86 and MHC-II molecules in CD11c^+^ BMDCs were significantly increased in the LPS (Lipopolysaccharide) and Neo-BCV groups, indicating that Neo-BCV can stimulate the maturation of BMDCs.

### 3.2. Anti-Tumor Efficacy of Neo-BCV

**Figure 2:**
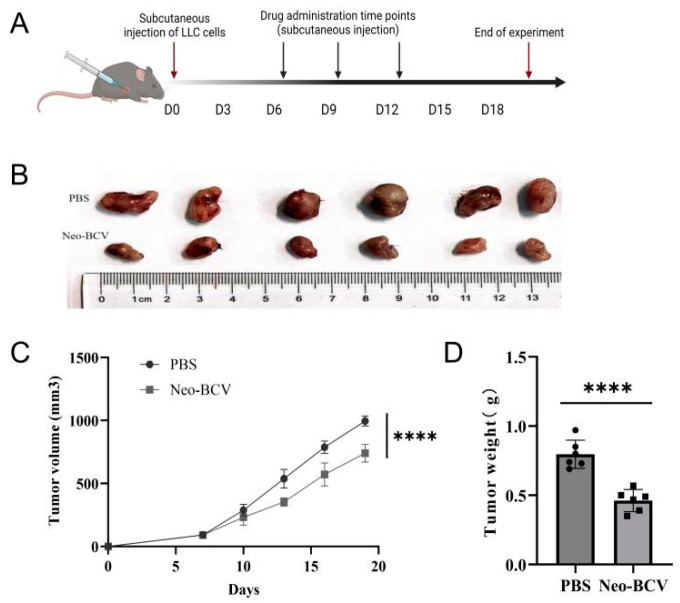
Neo-BCV vaccine induces tumor-specific immune responses in mice. (**A**) Neo-BCV experimental treatment protocol; (**B**) Images of mouse tumor tissues after treatment; (**C**) Tumor growth curves in mice; (**D**) Tumor tissue weights in mice after treatment. **** *p* < 0.0001.

C57BL/6 mice were injected with Neo-BCV or PBS subcutaneously twice per week after LLC subcutaneous implantation (Figure 2A). We determined the route and dose of administration of Neo-BCV after verifying the immunostimulatory response and safety of vaccination. On day 19 after tumor implantation, the mice were euthanized by cervical dislocation. Tumor, heart, liver, spleen, lung, and kidney tissues were harvested. Macroscopically, the tumors in the PBS group had an incomplete capsule and tended to adhere to the subcutaneous tissues, whereas the tumors in the Neo-BCV group had an intact capsule and hardly adhered to the subcutaneous tissues. The tumors were photographed, as shown in Figure 2B. Tumor volume was measured every three days, and the tumor growth curves of the mice are shown in Figure 2C. Tumor growth was rapid in the PBS group, while Neo-BCV significantly inhibited tumor growth. Tumor weight in the Neo-BCV group was significantly reduced compared to the PBS group (Figure 2D), with a tumor inhibition rate of 42%, indicating that Neo-BCV has a strong inhibitory effect on tumor growth.

### 3.3. Neo-BCV Mediates Activation of DCs and Induces Anti-Tumor Response of CTLs

**Figure 3:**
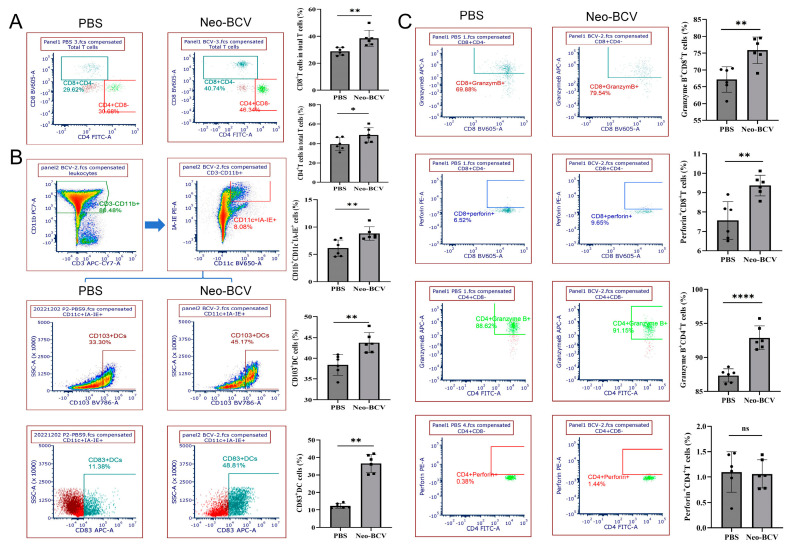
Immune response induced by Neo-BCV in the tumor. (**A**) Flow cytometry analysis of CD8^+^ T and CD4^+^ T lymphocyte expression in tumor tissues and semi-quantitative statistical charts. (**B**) Flow cytometry analysis of cDC1, CD103^+^ DC, and CD83^+^ DC expression in tumor tissues and semi-quantitative statistical charts. (**C**) Flow cytometry analysis of cytotoxic T lymphocyte perforin and granzyme B expression in tumor tissues and semi-quantitative statistical charts. n = 6, data are presented as mean ± standard deviation ( $\bar{x}$ ± s). Inter-group comparisons were analyzed using Student’s *t*-test. * *p* < 0.05, ** *p* < 0.01, **** *p* < 0.0001; ns indicates no significant difference.

To evaluate the immune activation capability of Neo-BCV in vivo, flow cytometry was used to further analyze the immune cells in the mice lung cancer tissues. In the tumor tissues, the infiltration of CD8^+^ T and CD4^+^ T lymphocytes in the Neo-BCV group was significantly higher than in the PBS group, as shown in Figure 3A. Similarly, the infiltration of dendritic cells (DCs) in the tumor tissue was analyzed. Type 1 conventional dendritic cells (cDC1, CD11b^+^ CD11c^+^ IA-IE^+^ cells) primarily function to present antigens to CD8^+^ T cells and initiate a Th1-type immune response, which is associated with favorable cancer prognosis [25,26]. As shown in Figure 3B, cDC1 infiltration in the Neo-BCV group was significantly higher than in the PBS group. Further analysis revealed that the expression levels of CD103 and CD83 on cDC1 in the Neo-BCV group were significantly higher than in the PBS group, indicating that the DCs in the Neo-BCV group have stronger antigen presentation ability, laying the foundation for an anti-tumor response.

T lymphocytes and NK cells can secrete Perforin and granzyme B. By contacting target cells, they release perforin and granzyme B, forming a channel on the surface of target cells, promoting their destruction [27,28,29,30]. As shown in Figure 3C, the percentage of CD8^+^ T lymphocytes secreting perforin and granzyme B, as well as the percentage of CD4^+^ T lymphocytes secreting granzyme B, in the Neo-BCV group was significantly higher than in the PBS group, with notable statistical significance.

In summary, the above results indicate that Neo-BCV can stimulate DCs to function, thereby mediating the anti-tumor activity of cytotoxic T lymphocytes.

### 3.4. Neo-BCV Improves the Spleen Immune Microenvironment

**Figure 4:**
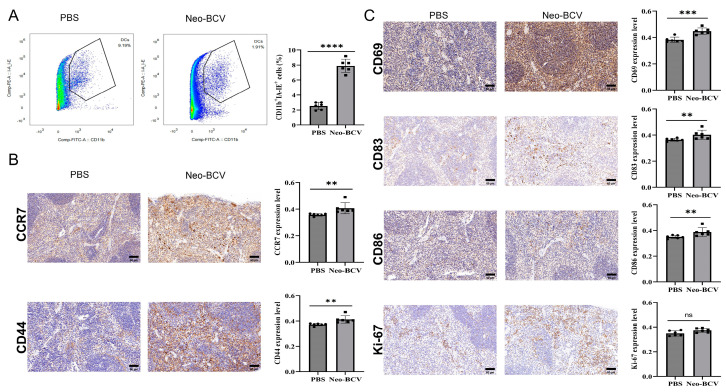
Immune markers induced by Neo-BCV in the spleen. (**A**) Flow cytometry analysis of DC expression in spleen tissues and semi-quantitative statistical charts. (**B**,**C**) Expression levels of CCR7, CD44, CD69, CD83, CD86, and Ki-67 in the spleen of the PBS and Neo-BCV-treated groups, along with semi-quantitative statistical charts. n = 6; staining intensity was used as the analysis metric, and statistical comparisons were made using the average OD value of the positive regions. Data are presented as mean ± standard deviation ( $\bar{x}$ ± s). Inter-group comparisons were analyzed using Student’s *t*-test; ** *p* < 0.01, *** *p* < 0.001, **** *p* < 0.0001; ns indicates no significant difference.

The spleen is an important lymphoid organ. Flow cytometry was used to assess DCs infiltration in the spleen, as shown in Figure 4A, where DCs infiltration in the Neo-BCV group was significantly higher than in the PBS group, with statistical significance. To further observe the immune infiltration in the mouse spleen, hematoxylin-DAB staining was used to detect the expression of CD44, CD69, CCR7, CD83, CD86, and Ki-67 in spleen tissue. The results, as shown in Figure 4B,C, indicate that the expression of CD44, CD69, CCR7, CD83, and CD86 in the Neo-BCV group was significantly higher than in the control group, while Ki-67 expression showed no significant difference between the two groups. CD44 is involved in various cellular functions, including T lymphocyte activation, recirculation, and homing [31]. CD69 plays a role in lymphocyte proliferation and serves as a signaling receptor in lymphocytes, natural killer cells, and platelets [32]. CCR7 mediates the directional movement and precise positioning of immune cells within lymph nodes [33]. CD83 and CD86 may play important roles in antigen presentation or cell–cell interactions after lymphocyte activation [34,35]. Ki-67 is mainly present in proliferating cells [36]. These findings suggest that Neo-BCV can alter the immune microenvironment of the spleen, promoting DC and T cell activation and inducing the expression of immune markers.

### 3.5. Study on the Anti-Tumor Immune Mechanism Mediated by Neo-BCV

**Figure 5:**
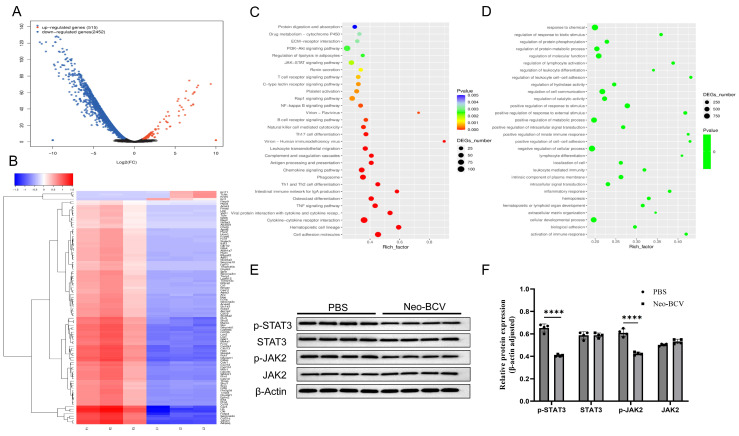
Tumor tissue transcriptomics.(**A**,**B**) Differential Gene Expression. (**A**) Volcano plot of differential genes, where the x-axis represents the fold change in gene or transcript expression between the two samples, and the y-axis represents the statistical significance of the differential expression, indicated by the *p*-value. (**B**) Clustering analysis of differential genes, with the color in the figure representing the expression level of that gene in this group of samples; red indicates high expression, while blue indicates low expression. (**C**,**D**) Enrichment Analysis of Differential Genes. (**C**) GO enrichment analysis, where each node represents a GO term, and the color intensity indicates the enrichment level; darker colors represent higher enrichment levels, with each node displaying the name of the GO term and the *p*-value from the enrichment analysis. (**D**) KEGG enrichment analysis, where the x-axis represents the enrichment factor and the y-axis represents the functional pathways enriched in the KEGG pathway. (**E**,**F**) Representative Western blot images and semi-quantitative statistical charts of the phosphorylation and total protein levels of STAT3 and JAK2 in tumor tissues. **** *p* < 0.0001. The original Western blot figures can be found in Supplementary File S1.

To further investigate the anti-tumor immune mechanisms mediated by Neo-BCV, we performed transcriptomic sequencing on the tumor tissues from the Neo-BCV and PBS groups. We then used the analysis software edgeR (Version 4.2.1) to conduct differential gene expression analysis, identifying 2967 genes with significant differential expression, of which 515 genes were upregulated and 2452 genes were downregulated, as shown in Figure 5A. A clustering analysis of the significantly different gene expression profiles was conducted, and the heatmap in the analysis results displays the top 100 genes with the lowest FDR values, as shown in Figure 5B.

To explore the pathways through which Neo-BCV activates DCs, we mapped the differential genes to the GO and KEGG databases for enrichment analysis, as shown in Figure 5C,D. The enrichment analysis identified pathways that were significantly enriched among the DEGs. The GO enrichment analysis indicated significant enrichment in pathways related to positive regulation of innate immune response, lymphocyte differentiation, leukocyte-mediated immunity, and intracellular signal transduction. The KEGG enrichment analysis revealed significant enrichment in pathways such as the JAK-STAT signaling pathway, NF-κB signaling pathway, antigen processing and presentation signaling pathway, chemokine signaling pathway, NK cell-mediated cytotoxicity, phagosome pathway, differentiation of Th1 and Th2 cells, and tumor necrosis factor signaling pathway.

Notably, the JAK-STAT signaling pathway was significantly downregulated in the Neo-BCV group and was one of the most enriched pathways. Numerous studies have shown that the JAK/STAT signaling pathway is closely related to the differentiation and maturation of DCs, making it worthwhile to explore further. To validate the results of the KEGG signaling pathways and clarify the molecular mechanisms by which Neo-BCV promotes DCs activation and maturation, we measured key proteins in the JAK2-STAT3 signaling pathway, as shown in Figure 5E,F. STAT3 is a key oncogenic factor in the JAK-STAT signaling pathway, and abnormal activation of STAT3 has been widely reported in various cancers, including lung cancer and hepatocellular carcinoma [37,38,39]. Our experiments found that after Neo-BCV treatment, only the phosphorylation levels of JAK2 and STAT3 in mice subcutaneous tumor tissues were significantly affected, indicating that Neo-BCV specifically inhibits the phosphorylation of JAK2, thereby suppressing the activation of STAT3. In summary, these results suggest that Neo-BCV acts as a JAK2 inhibitor, exerting anti-tumor effects by inhibiting the JAK2-STAT3 signaling pathway.

### 3.6. Safety Assessment of Neo-BCV

**Figure 6:**
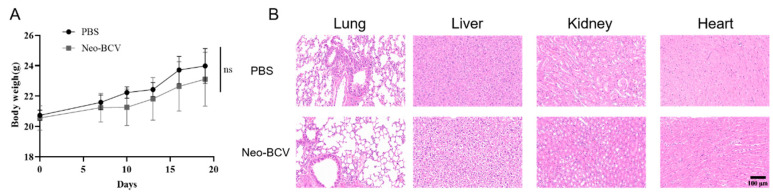
Safety considerations of Neo-BCV. (**A**) Body weight change curves of mice in different treatment groups. (**B**) H&E staining of major organs (lung, liver, kidney, and heart) after two weeks of different treatments (scale bar = 100 μm). ns indicates no significant difference.

Before and after tumor-cell implantation, the body weight of the mice was recorded every three days to observe changes in their weight. The excised heart, liver, lung, and kidney tissues were fixed in formaldehyde, embedded in paraffin, and stained with HE to observe tissue morphology and evaluate the in vivo safety of Neo-BCV. The changes in mouse body weight are shown in Figure 6A, indicating that the body weight of the Neo-BCV group was similar to that of the PBS group. The HE staining results of the lungs, liver, kidneys, and hearts of the mice are shown in Figure 6B, demonstrating that the morphology of the organs in the experimental group was similar to that of the PBS group, with no significant tissue lesions or abnormalities. This indicates that the novel bacterial liquid composite vaccine Neo-BCV has no significant toxic side effects and exhibits good in vivo safety.

## 4. Discussion

Lung cancer is one of the malignant tumors with an extremely poor prognosis. The traditional treatment methods for lung cancer include surgical resection, chemotherapy, and radiotherapy. In recent years, immune checkpoint blockade therapy has also gradually become popular. However, the effectiveness of these therapies is still relatively limited. Therefore, it is urgent to find new and more effective treatment methods.

Bacterial vaccines for treating tumors have a long history of development, with the milestone being the Coley toxin developed by William B. Coley, which successfully cured multiple patients with advanced cancer at that time.

In this study, we developed a novel bacterial composite vaccine (Neo-BCV) consisting of a variety of bacteria and toxins plus adjuvants. Unlike conventional specific anti-tumor vaccines, the anti-tumor effect of Neo-BCV is non-specific. Therefore, the Neo-BCV can be applied to a variety of cancer treatments, such as melanoma and pancreatic cancer, which are already under study. Our results indicate that Neo-BCV can improve the tumor immune microenvironment in mice and inhibit tumor growth in vivo. Specifically, Neo-BCV promotes the maturation and infiltration of DCs in the tumor sites. In-depth analysis shows that Neo-BCV promotes the differentiation of DCs into pro-inflammatory cDC1s, thereby activating the functions of CD4^+^ and CD8^+^ T and inducing their secretion of more effector factors and cytotoxic particles. Consistent with the tumor site, Neo-BCV also significantly improved immune infiltration in the spleen, indicating that it has a strong peripheral immune activation effect. Further mechanism research shows that the JAK-STAT signaling pathway was significantly downregulated in the Neo-BCV group, which is closely related to tumorigenesis, innate and adaptive immune responses. The results of WB show that the phosphorylation levels of JAK2 and STAT3 in the subcutaneous tumor tissues were significantly downregulated. These results indicate that Neo-BCV promotes T cell activation and function by inducing DCs differentiation and maturation, thereby exerting anti-tumor effects.

Numerous studies have shown that DCs play a significant role in regulating and shaping the anti-tumor immune system. As antigen-presenting cells (APCs), DCs play a crucial role in bridging innate and adaptive immune responses. The membrane and cytoplasmic receptors of DCs can recognize different types of danger signals, including pathogens and tumor cells. After antigen uptake and processing, activated DCs present it to immature T lymphocytes, triggering antigen-specific immune responses and exerting anti-tumor effects. DCs are considered a core component of the tumor microenvironment (TME). However, the immune desert microenvironment constructed by some tumors through known or unknown mechanisms can inhibit the function of DCs, alter their phenotype, and induce their differentiation into functional disorders and tolerance. Although immune checkpoint inhibitors (ICIs) have shown some effectiveness, only a small percentage of patients can benefit from them. In this article, our Neo-BCV effectively promotes the activation and maturation of DCs in tumor and spleen sites, inducing their differentiation into pro-inflammatory cDC1 (CD11b^+^ CD11c^+^ IA-IE^+^ cells), which drives us to pay attention to the relationship between them.

Current studies have pointed out that the JAK-STAT signaling pathway is closely related to tumorigenesis and innate and adaptive immune responses. The activation of the JAK/STAT pathway promotes the occurrence and progression of various diseases, including inflammatory diseases, lymphoma, leukemia, various solid tumors, etc. Among them, the JAK2-STAT3 signaling pathway is currently one of the main signaling pathways discovered. It participates in many physiological and pathological processes such as immune regulation, angiogenesis, cell proliferation, and differentiation. It is closely related to the occurrence, development, metastasis, and invasion of tumors, and its abnormal expression has guiding significance for tumor prognosis [33]. Based on this, we focused on studying the changes in this signaling pathway. Transcriptome and WB analysis show that the JAK-STAT signaling pathway is significantly downregulated in the Neo-BCV group. In-depth research has found that the phosphorylation levels of JAK2 and STAT3 are effectively inhibited. These results suggest that Neo-BCV acts as a JAK2 inhibitor, exerting anti-tumor effects by inhibiting the JAK2-STAT3 signaling pathway.

Although our study demonstrates the ability of Neo-BCV to improve the tumor immune microenvironment and inhibit tumor growth, there are still several limitations. First, the deeper connection between the JAK2-STAT3 signaling pathway and the differentiation and maturation of DCs has not been elucidated yet. Second, the regulatory effects of Neo-BCV on other immune cells besides DCs and T cells have not been studied. In addition, different tumor models, such as spontaneous tumor formation and tumor metastasis, need to be established to determine the universality of Neo-BCV to multiple tumor types.

Anyway, our findings suggest that Neo-BCV has great value in improving the tumor immune microenvironment of tumor patients clinically.

## 5. Conclusions

**Figure 7:**
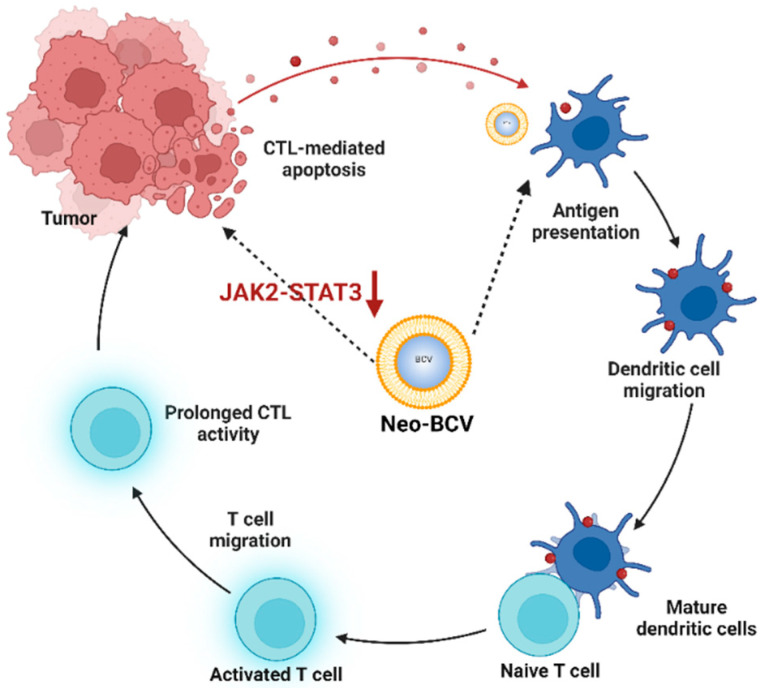
Summary diagram. Neo-BCV mediates CTL anti-tumor immune responses by activating /dendritic cells (DCs). Simultaneously, it exerts anti-tumor effects through the inhibition of the JAK2-STAT3 signaling pathway.

In conclusion, this study highlights Neo-BCV as a novel bacterial composite vaccine with significant potential in lung cancer immunotherapy. Neo-BCV effectively promotes the maturation and activation of dendritic cells (DCs), enhancing CD4^+^ and CD8^+^ T cell responses through DC-mediated antigen presentation (Figure 7). This activation of the immune system contributes to a favorable tumor microenvironment, marked by increased DC proportions in both tumor and spleen, which supports robust CTL-mediated anti-tumor effects. Transcriptomic analysis further confirms Neo-BCV’s unique ability to modulate the immune microenvironment by specifically inhibiting the JAK2-STAT3 signaling pathway, a key mechanism associated with innate and adaptive immune responses that has not been extensively explored in previous DCs-based cancer immunotherapy studies (Figure 7). While further investigation is needed to fully validate Neo-BCV’s long-term efficacy and safety in humans, our study establishes a strong foundation for future work in clinical application of cancer treatment.

## Data Availability

The original contributions presented in the study are included in the article. Further inquiries can be directed to the corresponding authors.

## Supplementary Materials

The following supporting information can be downloaded at: https://www.mdpi.com/article/10.3390/vaccines13010064/s1, File S1: The original Western blot figures.
